# Node Detection and Internode Length Estimation of Tomato Seedlings Based on Image Analysis and Machine Learning

**DOI:** 10.3390/s16071044

**Published:** 2016-07-07

**Authors:** Kyosuke Yamamoto, Wei Guo, Seishi Ninomiya

**Affiliations:** 1 Graduate School of Agricultural and Life Sciences, The University of Tokyo, 1-1-1 Midori-cho, Nishi-Tokyo, Tokyo 188-0002, Japan; kyosuke.yamamoto@15.alumni.u-tokyo.ac.jp (K.Y.); guowei@isas.a.u-tokyo.ac.jp (W.G.); 2 PS Solutions Corp., 1-5-2 Higashi-Shimbashi, Minato-ku, Tokyo 105-7104, Japan

**Keywords:** node detection, internode length estimation, image analysis, machine learning, BoVWs, affinity propagation

## Abstract

Seedling vigor in tomatoes determines the quality and growth of fruits and total plant productivity. It is well known that the salient effects of environmental stresses appear on the internode length; the length between adjoining main stem node (henceforth called node). In this study, we develop a method for internode length estimation using image processing technology. The proposed method consists of three steps: node detection, node order estimation, and internode length estimation. This method has two main advantages: (i) as it uses machine learning approaches for node detection, it does not require adjustment of threshold values even though seedlings are imaged under varying timings and lighting conditions with complex backgrounds; and (ii) as it uses affinity propagation for node order estimation, it can be applied to seedlings with different numbers of nodes without prior provision of the node number as a parameter. Our node detection results show that the proposed method can detect 72% of the 358 nodes in time-series imaging of three seedlings (recall = 0.72, precision = 0.78). In particular, the application of a general object recognition approach, Bag of Visual Words (BoVWs), enabled the elimination of many false positives on leaves occurring in the image segmentation based on pixel color, significantly improving the precision. The internode length estimation results had a relative error of below 15.4%. These results demonstrate that our method has the ability to evaluate the vigor of tomato seedlings quickly and accurately.

## 1. Introduction

In a tomato, vigor in the seedling stage determines the quality and final size of the fruit and the total production of the plant [1,2]. Thus, vigor in the seedling stage is an important trait in tomato breeding programs, and accurate evaluation of vigor is very important from a cultivation aspect. In general, vigor is evaluated by breeders or farmers using visual observation, which is time-consuming, labor-intensive, subjective, and sometimes inaccurate [3].

To overcome such problems, automated evaluation of plant vigor using image processing technology has become widespread in recent years [4,5,6,7,8]. The conventional method of image processing for extracting target information is based on the thresholding of features such as RGB color space [9], YCbCr color space [10], HSV color space [11,12], blob size [13], eccentricity [14], and texture [11]. However, the appearance of a plant dynamically changes by vigor and over the growing stage of the plant, making it necessary to adjust the threshold values for each image. Furthermore, as most plants are cultivated in fields or glasshouses, which involve varying lighting conditions and complex backgrounds, adjustment of threshold values is difficult. To overcome these problems, image processing technologies that employ machine learning have become increasingly available for field-based agronomic applications. In recent years, methods based on image processing technology and machine learning for the determination of the number of green apples [15], detection of immature peaches [16], extraction of vegetation area [17], extraction of tomato fruits including mature, immature, and young [18], and characterization of flowering dynamics in paddy rice [19] have been developed and are widely expected to be utilized in breeding programs and cultivation.

In tomato seedlings, characteristics such as number and size of leaves, height of seedlings, internode length, stem diameter, and dry matter content have been used as diagnostic indices [1,2]. In particular, internode length is sensitive to the salient effects of environmental stresses, including water shortage, high temperature at night, lack of sunlight, and excess nitrogen [20,21,22].

In fruit vegetables such as tomato, it is common to use grafting for the purpose of preventing soil borne diseases, improving tolerance for high/low temperatures, and increasing fruit quality and yield [23]. Generally, it is preferable for graft seedlings to have uniform internode length.

Because internode length is important in many types of plants, some research has been conducted on the detection of main stem nodes (henceforth called node) and estimation of internode length using image processing technologies. Davis [24] detected node regions from silhouette images of chrysanthemums via an artificial neural network using features calculated by the ring operator. Amean et al. [25] developed a method for extracting node regions from images of hibiscus nursery plants using a shape feature (the vesselness measure) and the Hough transform. McCarthy et al. [26] applied the same method to video sequences of cotton plants captured under field conditions. They compared sequential images in order to eliminate false positives and negatives. Although they tried to detect internode lengths based on their results, only 11% of the 840 internodes in the images were successfully detected, leaving much room for improvement. Despite these advances in developing methods for node detection and internode length estimation, no corresponding method for tomato plants has been proposed to date.

This study aimed to develop a method for internode length estimation using image processing technologies in order to diagnose the vigor of tomato seedlings. The resulting method involves three steps: node detection, node order estimation, and internode length estimation. In the first step, node regions are detected from the image of a tomato seedling via machine learning using local feature descriptors. In the second step, the node orders of the detected nodes are estimated with a non-hierarchical clustering method, i.e., affinity propagation [27], in order to estimate the internode lengths between adjoining nodes in the third step. The method has two main advantages: (i) as machine learning approaches are used for node detection, it does not require any adjustment of threshold values regardless of the images of different seedlings and allows for images to be taken at different timings and under various lighting conditions and with complex backgrounds; and (ii) as affinity propagation is used for node order estimation, it can be applied to seedlings with different numbers of nodes without first fixing the number of nodes as a parameter. We evaluated the performance of the method and its applicability for automated vigor diagnosis of tomato seedlings by detecting nodes and estimating internode lengths from time-series images of different seedlings.

## 2. Materials and Methods

### 2.1. Crop Materials and Image Acquisition

For this study, a tomato (*S. lycopersicum*) variety House Momotaro (Takii & Co., Ltd., Kyoto, Japan) was used. Tomato seedlings were grown in a glasshouse at the Institute for Sustainable Agro-ecosystem Services at the University of Tokyo ($35^{\circ }44^{\prime }09^{\prime \prime }$N and $139^{\circ }32^{\prime }27^{\prime \prime }$E). We used a hydroponic culture kit (Hyponica, Kyowa Co., Ltd., Osaka, Japan) for the culture. For image acquisition, a digital single-lens reflex camera (EOS 60D, Canon Inc., Tokyo, Japan) with an EF–S18–55 mm lens (Canon Inc., Tokyo, Japan) was set up in the glasshouse. The camera was controlled remotely in order to capture images at 8 p.m., 10 p.m., midnight, 2 a.m., and 4 a.m. on a daily basis. There were no other plants behind the target seedlings. Sowing dates, experimental period, and the number of captured images are shown in Table 1.

### 2.2. Node Detection

**Figure 1:**
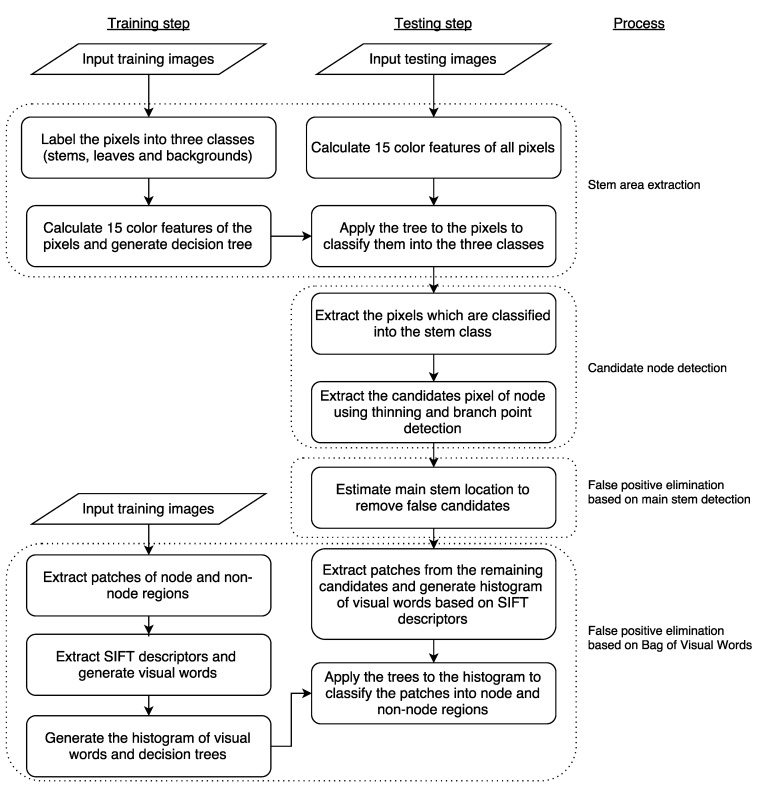
Flowchart of the developed method for node detection.

**Figure 2:**
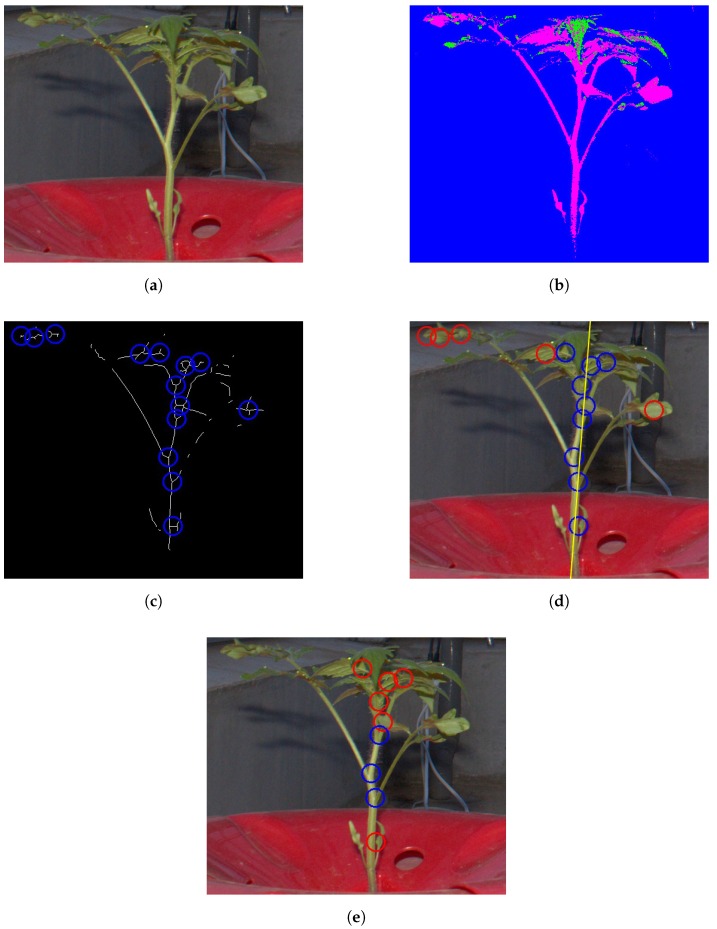
Example of node detection. (**a**) original image; (**b**) after pixel classification, where pixels drawn in green, purple, and blue were classified into leaf, stem, and background, respectively; (**c**) after thinning and branch point detection; (**d**) after main stem detection, where the yellow line represents the main stem line; (**e**) after Bag of Visual Words (BoVWs) application. In (**c**–**e**), the circle represents the candidate node pixels (blue: remaining candidate, red: removed candidate).

The method for node detection consists of four processes. A flowchart and application example of the developed method are shown in Figure 1 and Figure 2, respectively.

#### 2.2.1. Stem Area Extraction

In the first process, the images of the tomato seedlings are segmented based on pixel color using the decision tree-based segmentation model (DTSM) [17], which conducts plant segmentation based on the classification and regression tree (CART) classifier [28]. In the training step, we manually labeled pixels of training images into three classes: stems, leaves, and backgrounds. We acquired a total of 5000 pixels for the leaf class and 25,000 pixels for the stem and background classes as the training dataset. From the pixels, we then obtained 15 color features (r, g, b; H, S, V; $L^{*}$, $a^{*}$, $b^{*}$; $L^{*}$, $u^{*}$, $v^{*}$; Y, Cb, Cr) defined in five ordinarily used color spaces (RGB, HSV, $L^{*}$$a^{*}$$b^{*}$, $L^{*}$$u^{*}$$v^{*}$, and YCbCr). Finally, we build a decision tree based on the CART, which was tested by applying it to testing images in order to classify their pixels into the three classes (shown in Figure 2b).

#### 2.2.2. Candidate Node Detection

In this process, we extracted candidate node pixels. The value of pixels classified into the “stem” class in the first process was set to 1 (foreground), while other pixels were set to 0 (background). Hereinafter, we call this generated image the “stem image”. We then applied thinning [29] and branch and cross-point detection [30] to the generated image to extract candidate node pixels (shown in Figure 2c).

#### 2.2.3. False Positive Elimination Based on Main Stem Detection

The remaining processes were conducted to remove candidate pixels that were not close to any node. First, we extracted the connected region that stretched furthest along the *y*-axis in the stem images. Then, a linear regression of the *y*- and *x*-coordinate values in this region was conducted to obtain a line representing the approximate location of the main stem in the image (shown in Figure 2d). Hereinafter, this line is called the “main stem line”. As the nodes were located on the main stem, the candidate pixels of any candidate nodes located off the main stem line on the *x*-axis were removed as false positives. Based on a preliminary experiment, in this study, we set a fixed distance of 50 pixels, as all images were taken from the same distance.

#### 2.2.4. False Positive Elimination Based on Bag of Visual Words

**Figure 3:**
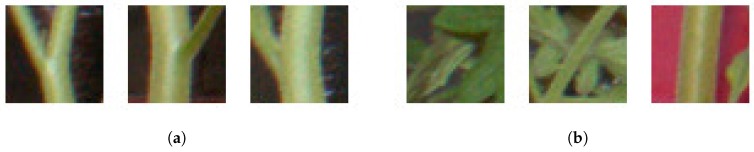
Examples of patches used for the training based on BoVWs. (**a**) node regions; (**b**) non-node regions.

Although the previous process removed most of the false candidates, it could not remove some false candidates from around the main stem. To remove these, we used an algorithm for generic object recognition, called “Bag of Visual Words (BoVWs)” [31]. In the training step, we manually extracted (40×40) pixel patches of node and non-node regions from the training images. Examples of such patches are shown in Figure 3, and the number of patches used for training is shown in Table 2. Then, the local feature points and descriptors of these points were extracted. In this study, we used the Harris corner detector [32] and Scale-Invariant Feature Transform (hereinafter referred to as SIFT) [33] for local feature point and descriptor extraction. Our targets, node regions of seedlings, have many corners, and thereby we choose the Harris for the local future points extraction. In general object recognition, SIFT has been widely used and its performance is already ensured, and therefore we used SIFT for local descriptor extraction. Then, visual words were generated based on *k*-means [34] with k=10. Histograms of visual words of the training images were then generated and used as training data for the random forest [35]. In the testing step, SIFT descriptors were calculated for local feature points extracted using the Harris corner detector from patches whose center of gravity was the candidate node pixel. If no feature point was extracted from a patch, it was identified as non-node region. Then, histograms of visual words were generated for each patch and used as an input to the random forest classifier. Finally, the center of gravity of the patches classified into the node region was used as the image node location. Default parameters in OpenCV [36] were used for Harris corner detection and SIFT descriptor extraction.

### 2.3. Node Order Estimation

**Figure 4:**
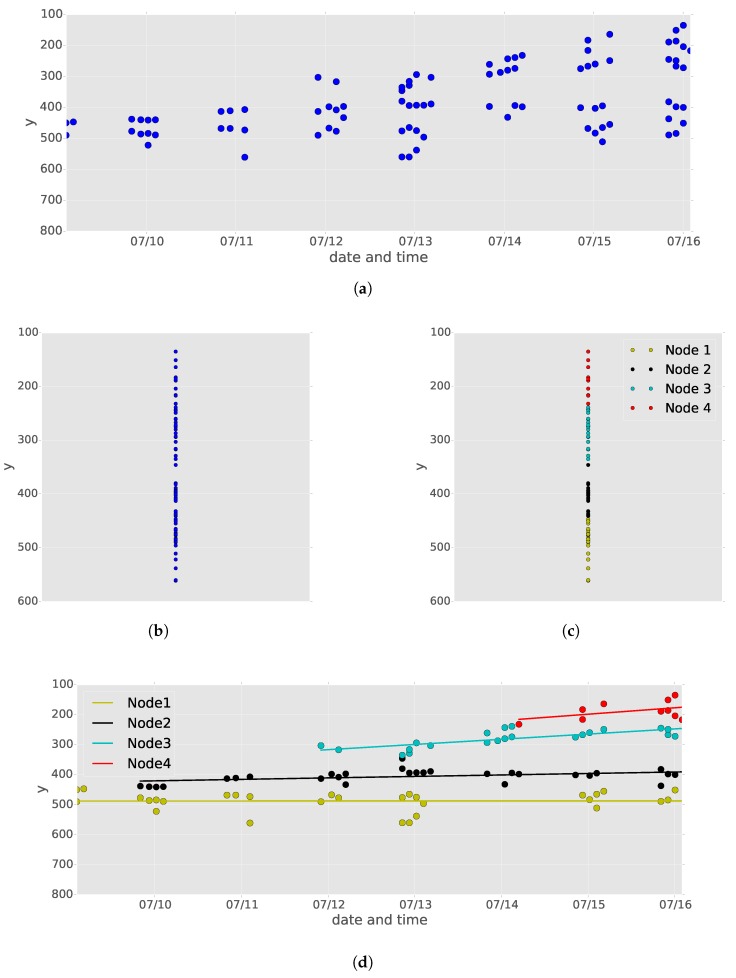
(**a**) typical results of node detection for time-series images of a tomato seedling; (**b**) after removing date and time information; (**c**) after applying affinity propagation, where data points with the same color belong to the same clusters; (**d**) after applying linear regression to each cluster.

A typical node detection result from time-series images of a tomato seedling is shown in Figure 4a. Because there are several false positives and negatives in the node detection results, it is very difficult to identify the detected node order in each image. However, the node order for each detected node must be determined in order to correctly estimate the internode length between adjoining nodes. In our method, we identified the detected node order by applying non-hierarchical cluster analysis and linear regression.

Figure 4 illustrates the procedure of node order estimation for detected nodes. First, we removed date and time information, which is represented on the *x*-axis, from the node detection results (Figure 4b). Then, affinity propagation [27] was applied, with damping factor=0.5 and the other parameters were set to default values in the “apcluster” package for R [37] to classify the detected nodes into *n* classes according to their *y*-coordinate values (Figure 4c). In this study, similarity between input data points is set to a negative squared distance.

Affinity propagation, one of the primary non-hierarchical clustering methods, has some advantages over other methods in determining the number of clusters. In *k*-means, a typical non-hierarchical clustering method, the number of clusters *k* must be provided by the user in advance. However, in our case, the number of clusters is unknown because the number of nodes differs by seedling. In affinity propagation, by contrast, the optimal number of clusters *n* is automatically determined by passing messages between data points of the input dataset. Briefly, the exemplar of the clusters (and for the other data points, which exemplar they belong to) is determined based on two messages: responsibility and availability. The responsibility, which is sent from data points to candidate exemplars, determines how appropriate an exemplar is for having a data point belong to it. The availability, which is sent from candidate exemplars to the other data points, determines how appropriate it is for a data point to belong to an exemplar. As a result, we can expect that the number of clusters determined by affinity propagation becomes the same as the number of nodes in each seedling.

From the results of affinity propagation, we set node orders from 1 to *n* to generate clusters in decreasing order of the means of the *y*-coordinate values of the clusters. Finally, we recovered the date and time information for all data points and then applied linear regression of *y* given the date and time information (UNIX time) of the data points in each cluster (Figure 4d). The resulting *n* lines, which are called “node lines” in this paper, were used to determine the *y*-coordinate values of the nodes for each node order at any date and time.

### 2.4. Internode Length Estimation

We obtained the node lines, which determine the location of nodes in an image, by applying the above method for node order estimation. We defined the internode length between the *i*-th and (i+1)-th nodes as a distance between the *i*-th and (i+1)-th node lines on the *y*-axis at each time. The internode length between the *i*-th and (i+1)-th nodes was calculated only after the (i+1)-th node appeared. We converted the pixel-based distance into a real quantitative measure (mm) based on an index object, whose actual size is known, in the images. Based on a preliminary experiment, we defined the relation between the pixel-based distance and the real quantitative measure as 0.41 mm/pixel.

### 2.5. Performance Evaluation

We conducted leave-one-seedling-out cross validation using time-series images of one seedling obtained for training and those of the others for testing, repeating this for all possible combinations among three seedlings in order to evaluate the performance of the proposed method for node detection and internode length estimation.

To evaluate the performance of the node detection method, two indices, recall and precision, were used. They are defined as follows:

(1) $$\mathrm{Recall}=\frac{\text{The number of relevant detections in images (TP)}}{\text{The actual number of nodes in images (TP + FN)}}$$

(2) $$\mathrm{Precision}=\frac{\text{The number of relevant detections in images (TP)}}{\text{The number of detections in images (TP + FP)}}$$

where TP, FP, and FN represent true positive, false positive, and false negative, respectively. We manually extracted true node regions as ground truth with rectangle ranged 28–80 and 28–82 pixels in width and height. Detected node regions were counted as TP if its center of gravity was within the true node regions. Recall is the measure of completeness, and precision is the measure of exactness. These indices were calculated using the results of node detection (i) without applying any method for false positive elimination; (ii) while applying main stem detection; and (iii) while applying main stem detection and BoVWs based on the leave-one-seedling-out cross validation.

Relative error, which is defined as Equation (3), is used to evaluate the internode length estimation method:

(3) $$\mathrm{Relative}\;\mathrm{error}\;(\%)=\frac{|\sum_{i=1}^{N}\hat{l}-\sum_{i=1}^{N}l|}{\sum_{i=1}^{N}l}\times 100\%$$

where *l* and $\hat{l}$ represent, respectively, the observed and predicted internode lengths between adjoining nodes in time-series images, and *N* represents the number of time-series images of a seedling. Relative error was calculated for each internode.

### 2.6. Implementation

Image processing using procedures such as color space transformations and local feature point and descriptor extractions were conducted using OpenCV [36] with Python 2.7. Thinning and branch point detection were performed using the “scikit-image” package for Python. To implement the machine learning methods, CART, *k*-means, and affinity propagation were performed using R version 3.2.3 (R Core Team, Vienna, Austria) [37].

## 3. Results

Table 3 describes the results of the node detection tests based on leave-one-seedling-out cross validation. The proposed method could detect 72% of the 358 nodes in the time-series images of all seedlings (recall=0.72). In addition, 78% of the detected regions were relevant to the node regions observed by visual inspection (precision=0.78), demonstrating that the accuracy of the developed method was quite high. The best result was obtained from seedling C (recall=0.91,precision=0.91), while the worst result was obtained from seedling B (recall=0.61,precision=0.66). There was no trend indicating whether a specific node order provides better or worse results.

**Figure 5:**
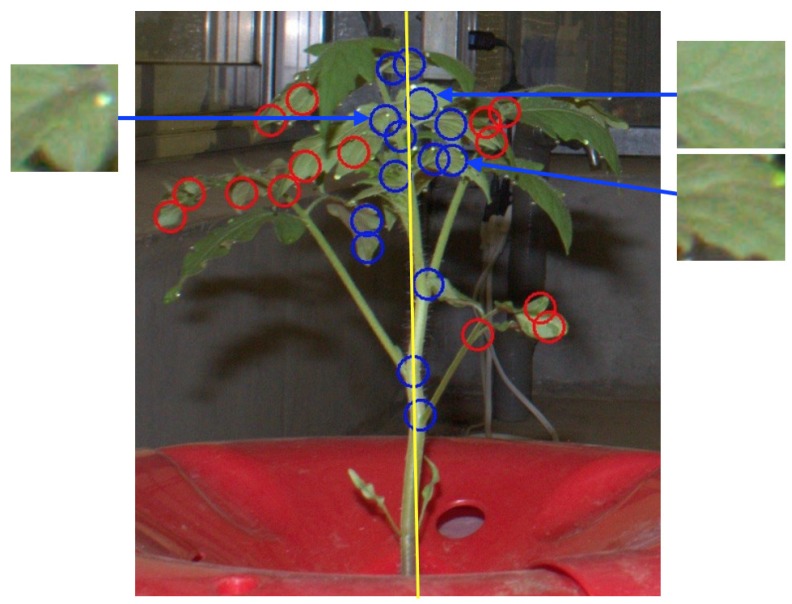
False positives occurring on a leaf. The circles represent the candidate pixels of the node after applying main stem detection (blue: remaining candidate, red: removed candidate). The yellow line represents the main stem line.

Precision was significantly improved by applying false positive eliminations based on main stem detection and BoVWs. In particular, BoVW application increased precision from 0.37 to 0.78, corresponding to the elimination of a very large number of false positives. This occurred because, as shown in Figure 5, many false positives occurring on leaves around the main stems were not eliminated by the main stem detection method. However, as the texture of a leaf is more complicated than that of a node, they were properly removed by BoVWs. On the other hand, recall was decreased from 0.77 to 0.72 by applying the two methods for false positive elimination. This suggests that some of the detected node regions relevant to the observed node regions were also removed. The node order estimation and internode length estimation were conducted based on the result of the node detection using the main stem detection and BoVWs.

**Figure 6:**
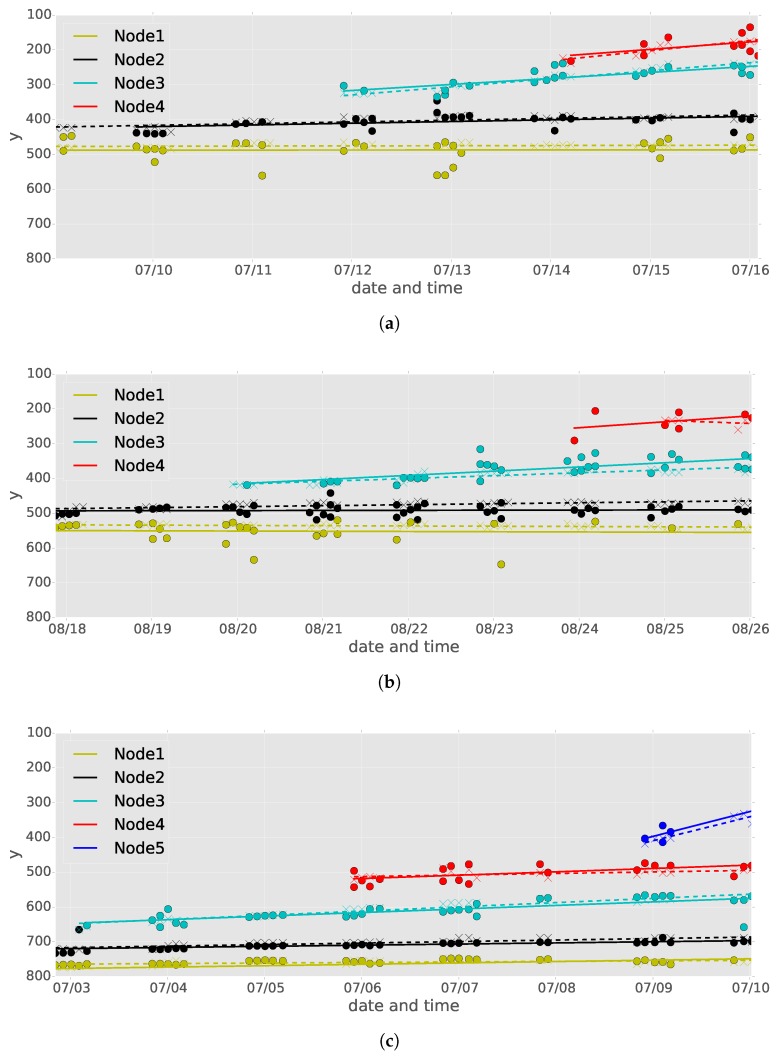
Result of node order estimation of (**a**) seedling A, (**b**) seedling B, and (**c**) seedling C. The horizontal and vertical axes represent the image capturing date and time and *y* coordinate value of the detected node, respectively. ∘ and × represent the detected and observed nodes, and solid and dashed lines represent the node lines calculated on the basis of detected and observed nodes, respectively. Points and lines with the same color belong to the same node orders.

The result of node order estimation is shown in Figure 6. The solid node lines calculated on the basis of the detected nodes are very close to the dashed node lines calculated on the basis of the observed nodes, confirming that node order estimation was conducted accurately. In this paper, we call the former lines “detected node lines” and the latter ones “observed node lines”.

As shown in Figure 6, the *y* coordinate values of the detected nodes fluctuated with time. In particular, the fluctuations in the first node of seedlings A and B were larger than those in the others. This occurred because some cotyledonary nodes were also detected by the proposed node detection method. Despite these false detections, application of linear regression canceled the effects of the false detections, resulting in detected node lines that were close to the observed node lines. On the other hand, there were many false negatives in the first node of seedling B after August 22nd. However, this node was detected before and after the time the false negatives occurred, allowing the false negatives to be interpolated by linear regression.

During the experimental period, four nodes appeared on seedlings A and B and five nodes appeared on seedling C. Affinity propagation, which automatically determines the best number of clusters based on the distribution of an input dataset, generated four clusters on seedlings A and B and five clusters on seedling C.

The results of estimation of internode length are shown in Table 4. The relative errors of all internodes were below 15.4%. The maximum relative error was observed between the second and third nodes of seedling B (38.6%), while relative error between first and second nodes of seedling B was the smallest (0.4%) in internodes of the seedlings.

## 4. Discussion and Conclusion

This paper proposes an image processing method to accurately detect nodes and estimate internode length using a conventional RGB digital camera in conjunction with machine learning approaches. This is the first study on node detection and internode length estimation from tomato seedling images taken in a condition close to actual cultivation. Because the proposed method is conducted using computers, it can evaluate internode length more efficiently than conventional visual inspections.

The method developed for node detection consists of four processes: image segmentation at a pixel level, detection of candidate pixels for a node, false positive eliminations based on main stem detection, and false positive eliminations based on BoVWs. The first and fourth processes were conducted based on classification models generated by machine learning approaches, and, therefore, do not require an adjustment of threshold values for different seedlings. On the other hand, in the third process, we applied thresholding of the distance from the main stem line to the node candidate pixels. We used the same threshold values for all seedlings in this study because all images were taken from the same distance. In cases where images taken from different distances are used, we plan to measure the distance using depth sensors or stereo vision to determine the threshold values.

In our proposed method for node detection, we first applied image segmentation on a pixel level to extract stem pixels, to which the subsequent methods were applied. Thus, if all pixels of a node were to be misclassified, we would not have been able to detect the node. In short, the accuracy of our method for node detection strongly depends on the accuracy of DTSM. In Guo et al. [17], DTSM could extract 80% of the vegetation regions from wheat images taken in a field. However, we did not evaluate the accuracy of DTSM quantitatively, and this must be done in a future study. On the other hand, there is a method for avoiding the application of DTSM: the sliding-window approach [19,38,39], in which an image is scanned using a small window to evaluate whether the window contains nodes. However, the sliding-window approach increases the computation cost exponentially and its accuracy strongly depends on the window size, which must be predefined by the user.

We approximated the path of the main stem as a line. This approach was successful because the stems of all seedlings used in this study were straight and elongated. However, it is known that stems elongate along a line that curves depending on the cultivation conditions. In such cases, the distance between the nodes and the main stem line increases, and therefore, the threshold value of the distance must be adjusted depending on the form of stem elongation. To solve this problem, we plan to adjust the threshold value of the distance based on the $R^{2}$ values; for stems elongating along a curved line, $R^{2}$ will have a low value, and, therefore, the threshold value should be larger than that in the case of a straight stem. Instead of linear regression, curve fitting of the main stem also might solve such problem. This approach allows to measure the two-dimensional internode length, while this study considered the internode length only in *y*-axis. In addition, stems do not grow exactly parallel to the imaging plane, and thus we also plan to use 3D curve fitting to measure internode length of curved stem properly, introducing 3D structure measurement methods.

Generic object recognition remains as a popular research topic and continues to be developed. There have been many improvements on BoVWs so far [40,41,42,43]. According to Chatfield et al. [44], the Fisher vector developed by Perronnin et al. [43] performs the best among recent algorithms. Wang [45] and Long [46] used affinity propagation, which is used for the node order estimation in the current study, to generate visual words in BoVWs. Meanwhile, deep learning [47] has received considerable attention in recent years. Deep learning surpassed BoVWs in terms of accuracy of object recognition [48] in the ImageNet Large Scale Visual Recognition Challenge 2012 [49], and, hence, the utilization of such technology may increase the accuracy of node detection in future research.

The number of leaves of a tomato plant increases as it grows. Because leaves hide nodes, the method developed here for node detection is applicable only to tomato plants in the beginning the seedling stage. Because we apply DTSM in the first stage of node detection, we can extract not only stem regions but also leaf regions accurately. This information will help in predicting the frequency of node occlusion by leaves, and we expect to predict the accuracy of node detection based on the amount of leaves in order to enable omission of images to which our method is not applicable.

In the node order estimation, the numbers of clusters determined by affinity propagation were equal to the number of nodes of each seedling. Thus, our method was applicable to seedlings with different numbers of nodes without parameter optimization. This result indicates that our method can be utilized even in a tomato field in which seedlings with different numbers of nodes are grown. In addition, the application of linear regression successfully (i) minimized the negative effects of false positives on internode length estimation; and (ii) enabled interpolation of false negatives based on time-series information.

**Figure 7:**
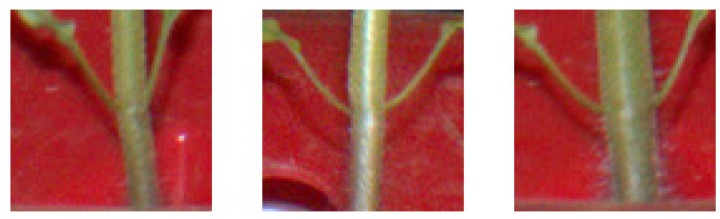
Examples of cotyledonary node regions.

Relative error for internodes between the second and third nodes of seedling B was extremely high compared to those for the other internodes. Such high relative error was caused by the detection of cotyledonary nodes. In the node order estimation based on affinity propagation, cotyledonary nodes with extremely high *y*-coordinate values were included into the cluster of the first node. Thus, some of the detected nodes with relatively high *y* coordinate values in the detected second nodes were classified into the cluster of the first node on around 21 August. In addition, there were many false positives between the third and fourth nodes that were classified into the cluster of the third node. As a result, the *y*-coordinate values of the second and third nodes were overestimated and underestimated, respectively, and, therefore, the internode length between the nodes was overestimated. Especially toward the former issue, we plan to use the patches of cotyledonary node regions, which were used as non-node region in this study, as cotyledonary node region in future studies. As shown in Figure 7, cotyledons are different in their symmetrical structure and the thickness of the cotyledonary petioles from the node regions shown in Figure 3a. Therefore, the cotyledonary nodes can be easily eliminated.

The relative errors of internode length estimation between the *i*-th and (i+1)-th nodes do not depend on the recall of node detection of the nodes. This is because we used linear regression, which makes the detection of all nodes in all images unnecessary. If a node is detected in some of the images, the location of the node in the other images will be interpolated by linear regression. On the other hand, many false positives decrease the accuracy of node order estimation. For this reason, we should try to increase precision rather than recall in node detection in order to increase the accuracy of internode length estimation. Therefore, we should only detect node regions with high degrees of certainty, while the others should be classified as non-node region, especially in processes based on BoVWs. We hope to use methods such as weighted random forest and balanced random forest, which were developed by Chen et al. [50] and are able to set weighting coefficients for each cluster, to extract node regions with a high degree of certainty.

The number of samples used in this study was insufficient to prove the versatility of our method. In fact, it is known that internode lengths are changed by cultivation conditions; in particular, low night temperatures or water shortages will shorten internode lengths [20,21,22]. In such cases, it would be difficult to apply our node order estimation because nodes of different node order may overwrap in time-series. It is very important to evaluate whether our method is applicable in such cases in a future study. We may solve such case by dividing time-series images into several groups and applying affinity propagation to group by group. In this approach, how to divide the time-series images need to be considered. For instance, we are considering the following methods: (i) dividing into the equal size of groups; (ii) using sliding-window approach; and (iii) finding the best size of groups based on the minimization of the difference of *n* among the groups.

In this study, we used images of tomato seedlings taken at times in which the effect of sunlight was low. The importance of using images taken in the daytime seems to be low because the stem of a tomato plant elongates more in dark periods than in photoperiods [51]. However, Shimizu and Heins [52] reported that the difference between the temperature in a photoperiod (DT) and a dark period (NT), commonly referred to as DIF, controls stem elongation during a photoperiod. Therefore, it may be necessary to measure internode lengths in both photoperiod and dark period.

Research centered on trying to control the internode length for better grafting using environmental factors was carried out by Koyano et al. [53]. The method proposed in this study will accelerate such research by enabling the automated measurement of internode length, which was measured manually in Koyano et al. [53]. Our method can also be utilized in horticultural research. The length of a cut flower, which is an important index for evaluating the quality of a commodity, is mainly determined by the number of nodes and the internode length. In particular, internode length is a key index because it can be controlled through environmental management. In general, cut flowers have fewer leaves than fruit vegetables, and correspondingly, we expect to achieve higher accuracy for cut flowers than for fruit vegetables in both node detection and internode length estimation.

In this study, we used images of tomato seedlings taken with a fixed-point camera. However, information obtained using one fixed-point camera is not enough to provide sufficient information to farmers or breeders. Plant factories, especially in Japan, have equipment for running self-propelled vehicles. The mobile robot field server developed by Fukatsu [54] can navigate a field and capture crop images. In future work, we hope to use such hardware to cover a large number of seedlings. This approach will open the way to precision agriculture based on image processing technologies.

## Figures and Tables

**Table 1:** Sowing dates, experimental period and the number of captures images.

Seedling	Sowing Date	Experimental Period	Number of Images
A	19 June 2014	9–16 July 2014	36
B	23 July 2014	18–26 August 2014	43
C	19 June 2014	2–10 July 2014	35

**Table 2:** Number of patches used for training based on Bag of Visual Words (BoVWs).

	Seedling A	Seedling B	Seedling C
Node region	212	191	211
Non-node region	242	286	255

**Table 3:** Recall and precision of node detection with and without false positive elimination (Original: without any false positive elimination, MS: false positive elimination based on main stem detection described in Section 2.2.3, MS + BoVWs: both false positive eliminations based on main stem detection and BoVWs described in Section 2.2.3 and Section 2.2.4.).

Seedling	Node Order	Original		MS		MS + BoVWs		Total Node Number
		Recall	Precision	Recall	Precision	Recall	Precision	
A	1	0.72	-	0.72	-	0.61	-	36
	2	0.89	-	0.89	-	0.69	-	36
	3	0.83	-	0.83	-	0.74	-	23
	4	0.64	-	0.73	-	0.45	-	11
	All	0.79	0.21	0.80	0.42	0.64	0.79	106
B	1	0.67	-	0.65	-	0.58	-	43
	2	0.67	-	0.65	-	0.63	-	43
	3	0.41	-	0.41	-	0.59	-	32
	4	0.67	-	0.50	-	0.50	-	6
	All	0.63	0.24	0.61	0.35	0.61	0.66	124
C	1	0.94	-	0.94	-	0.94	-	35
	2	1.00	-	1.00	-	0.94	-	35
	3	0.94	-	0.97	-	0.97	-	32
	4	0.68	-	0.68	-	0.79	-	19
	5	0.71	-	0.57	-	0.43	-	7
	All	0.92	0.17	0.92	0.40	0.91	0.91	128
All	All	0.77	0.17	0.76	0.37	0.72	0.78	358

**Table 4:** Result of internode length estimation.

Seedling	Internode	Mean of Internode Length		Relative Error (%)
		Observed (mm)	Estimated (mm)	
A	1–2	29.6	33.1	11.5
	2–3	47.2	49.2	4.3
	3–4	24.3	27.7	13.9
	All	34.6	37.5	8.5
B	1–2	25.0	25.1	0.4
	2–3	33.2	46.0	38.6
	3–4	55.3	49.8	10.0
	All	30.5	35.2	15.4
C	1–2	23.2	22.7	2.3
	2–3	39.6	40.1	1.4
	3–4	35.0	40.0	14.2
	4–5	48.5	49.1	1.2
	All	33.1	34.2	3.2
